# Bent Intramedullary Femoral Nail: Surgical Technique of Removal and Reconstruction

**DOI:** 10.1155/2011/614509

**Published:** 2011-10-12

**Authors:** Vasileios I. Sakellariou, Stamatis Kyriakopoulos, Helias Kotoulas, Ioannis P. Sofianos

**Affiliations:** ^1^1st Department of Orthopaedics, Athens University Medical School, University of Athens, ATTIKON University General Hospital, 1 Rimini Street, Chaidari, 12462 Attica, Greece; ^2^Department of Orthopaedics, General Hospital of Levadia, 1 Agiou Vlassiou Street, 32100 Levadia, Greece

## Abstract

A secondary high-velocity trauma to a previously stabilized femoral fracture with intramedullary nailing is rare. In this paper, we present the management of a 40-year-old man presented with a bent intramedullary nail due to secondary trauma. A lateral longitudinal femoral osteotomy was used for the resection of the distorted nail. The femur was reconstructed with a new nail, and the fixation of the osteotomy was achieved with plate and cerclage wires. Five months postrevision surgery, callus formation was evident and the patient regained a normal range of motion and gait, walking with a single cane.

## 1. Introduction

A secondary high-velocity trauma to a previously stabilized long bone fracture with an intramedullary nail is very rare. No standard protocol exists in the literature as very few cases have been documented [1–5]. 

The purpose of this paper is to present the management of a case where an intramedullary nail, placed to fix a right femoral shaft fracture, was distorted (bent to about 130°) 13 months postoperatively, due to a secondary trauma. 

## 2. Material and Methods

A 40-year-old male patient, known to be a drug abuser, HCV positive, and a heavy smoker, was admitted to our department after suffering a car accident with an open Gustillo II type fracture of the right femur. An open reduction and internal fixation were performed using a D.C.P. plate and 4.5 mm screws, as there was not an off-the-shelf availability of intramedullary nails or external fixators at the time of injury. The operation was performed few hours after admission and did not end to a stable fixation.

**Figure 1:**
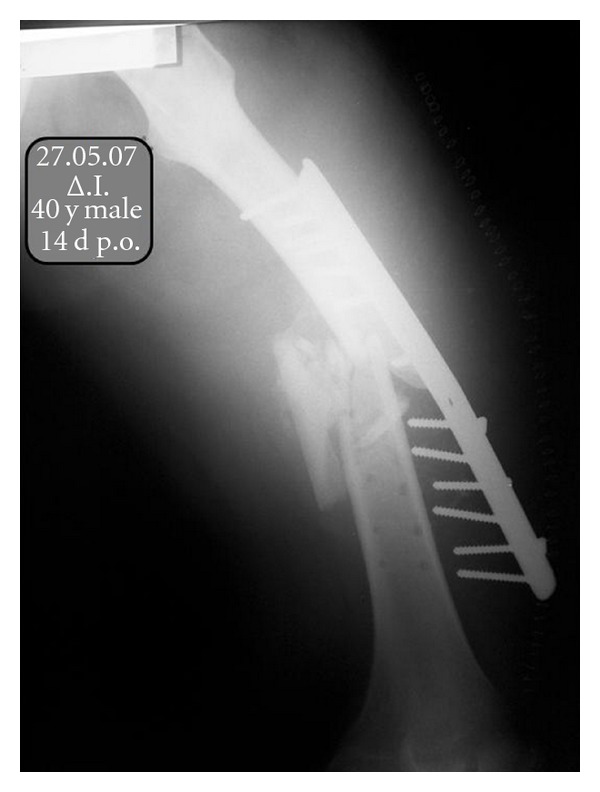
Mechanical failure of the initial internal fixation two weeks postoperatively.

Two weeks postoperatively, a mechanical failure of the internal fixation was observed due to suboptimal fracture fixation (Figure 1). 

**Figure 2:**
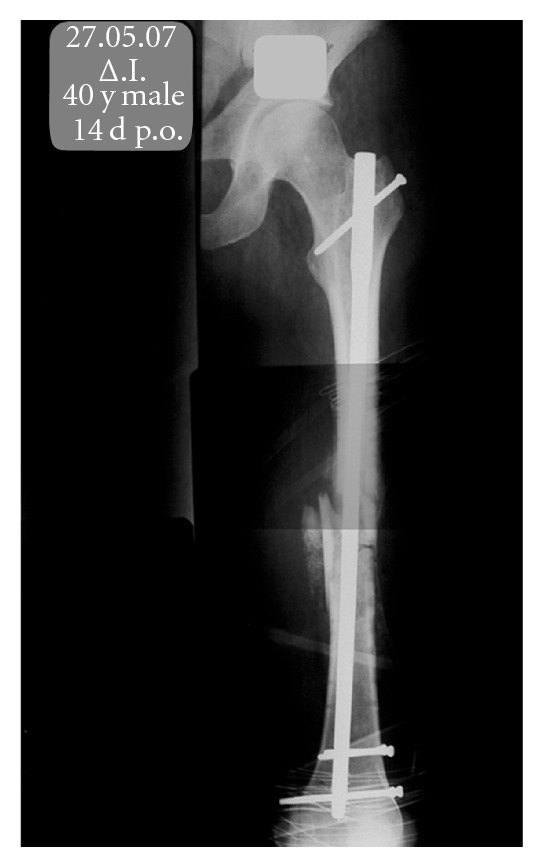
Revision of initial internal fixation with static intramedullary nailing and bone allografts.

The patient returned to the operating room for the replacement of the plate and screws with an interlocking nail. Bone allografts were also placed on the fracture site (Figure 2). 

Four months postoperatively, there was no satisfactory evidence of bone healing, but we decided to remove the distal screws of the nail because the patient already started weight bearing arbitrarily, and, as a result, one of the screws was broken and the other distorted. DBM enhanced by mixing with aspirated bone marrow (Ignite, Wright Medical Technology, Inc., Arlington, Tenn, USA) was placed on the fracture site. The patient was subsequently instructed to progressively achieve full weight bearing abilities within a six-week period. At the monthly followup, radiographic and clinical images showed a satisfactory rate of callous bone formation. Three to four months later, we lost regular contact with the patient.

**Figure 3:**
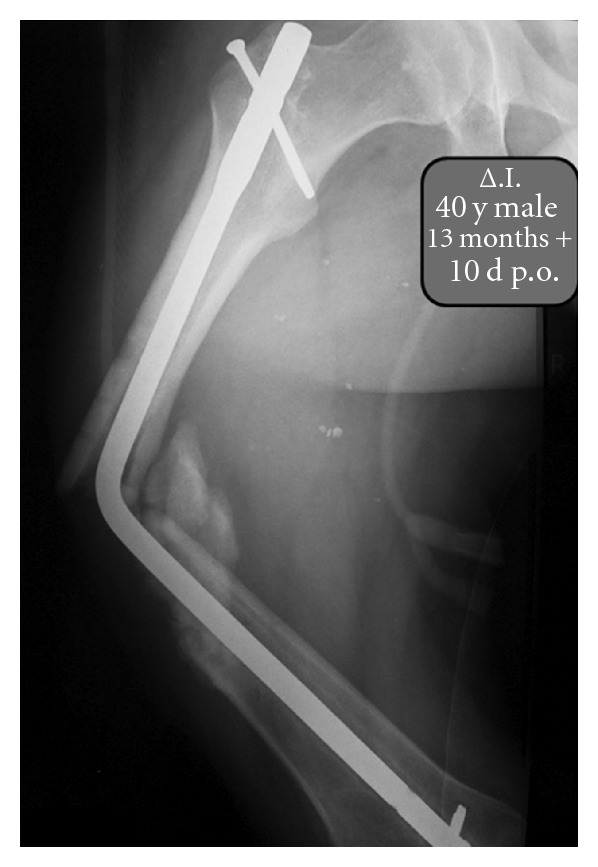
Radiographic image showing refracture of the femoral shaft and a bending deformation of the intramedullary nail.

Approximately 13 months postintramedullary nailing, the patient was involved in another car accident and was admitted to our department due to pain and deformation of the femur in recurvatum. Plain radiography revealed a refracture of the femoral shaft and a bending deformation of the intramedullary nail (Figure 3). 

**Figure 4:**
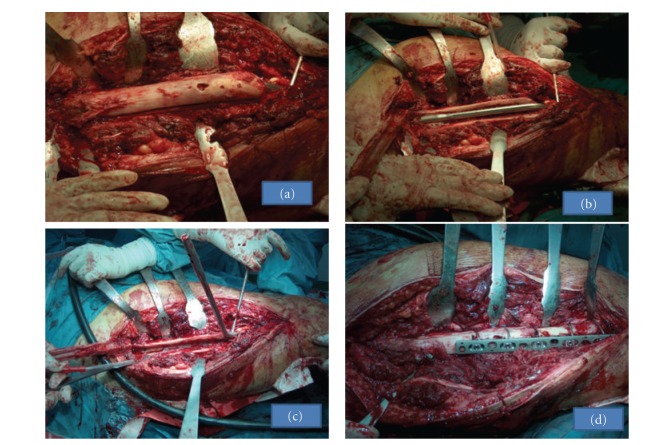
Surgical technique. (a) A proper osteotomy was performed as to facilitate the creation of a longitudinal bone window along the anterolateral side of the distal part of the femoral shaft. The osteotomy extended from the fracture site distally to the greater trochanter. (b) Anterolateral window of the femoral shaft approximately 2 cm in width. (c) Removal of the intramedullar nail through the window. (d) Reattachment of the bone window, after the placement of a new reconstruction nail, which was secured by a buttress plate (Accord) and cerclage wires.

At the preoperative evaluation, it was considered impossible to straighten out the nail or to cut it so as to facilitate its removal. After general anesthesia and despite our best attempts, this proved to be true. Therefore, we continued to perform a proper osteotomy as to facilitate the creation of a longitudinal bone window along the anterolateral side of the distal part of the femoral shaft. The osteotomy extended from the fracture site distally to the greater trochanter proximally, approximately 2 cm in width. Subsequently, we easily removed the nail and replaced it with a new one. The osteomized bone strip window was secured with a special buttress plate (Accord, Smith & Nephew, Inc., Memphis, Tenn, USA) stabilized by cerclage wires (Figures 4(a)–4(d)). 

As it was certain that the nail was well fixed in place and as the operation time was quite extended, it was decided to temporarily postpone the distal locking of the nail. A large amount of DBM mixed with cancellous bone allografts was placed on the fracture site and alongside the lateral longitudinal osteotomy. 

## 3. Results

The postoperative radiographs showed a good reduction both in the coronal and sagittal planes. A secure fixation was also observed. Subsequently, the wound healed without further complications.

**Figure 5:**
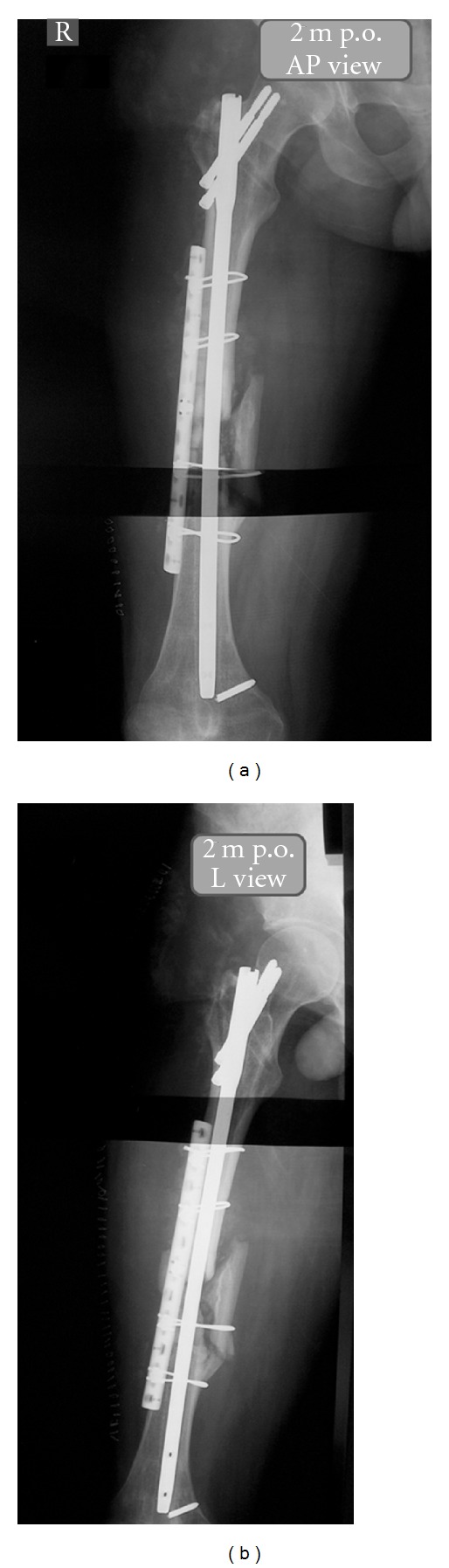
Radiographic imaging of femoral reconstruction two months postoperatively in (a) anteroposterior and (b) lateral view, showing satisfactory callus formation of both the fracture and the osteotomy of the femur.

Two months after revision, the patient was fitted with a lumbar femoral tibial splint. Furthermore, a nonweight-bearing crutch walking has been initiated. Radiographs reveal good evidence of bone reaction and callus formation and a general healing progression (Figures 5(a) and 5(b)). 

**Figure 6:**
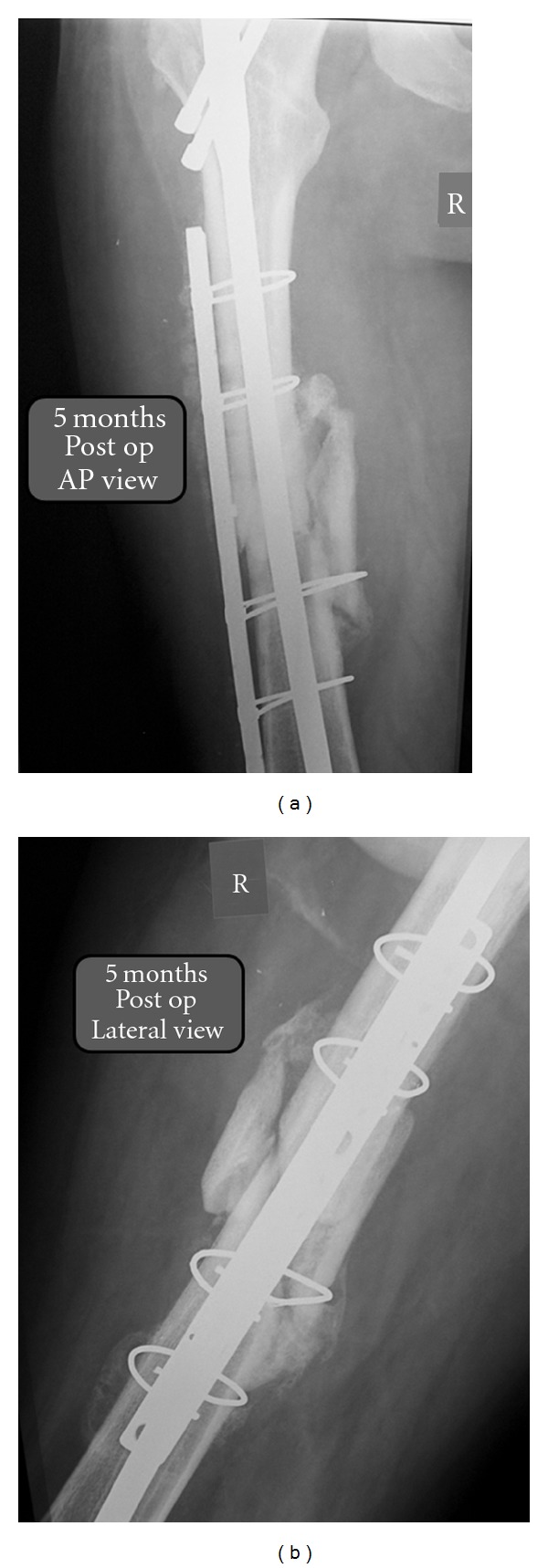
Radiographic imaging of femoral reconstruction five months postoperatively in (a) anteroposterior and (b) lateral view, showing progression of callus formation.

Five months after revision, the splint has been removed and the patient has been instructed to start weight bearing progressively. Subsequent radiographs revealed continued evidence of callus formation (Figures 6(a) and 6(b)). 

Twenty four months after revision, the patient has regained full range of motion of the hip and knee, without abductor mechanism deficiency. The gait has returned to normal without any need for additional supporting devices. No clinical, radiographic, or laboratory findings of periprosthetic loosening or infection have been identified, while bone healing and callus formation are satisfactory. 

## 4. Discussion

The use of intramedullary nails for the treatment of femoral fractures is the gold standard [6]. Less comminution of fractures is observed when the intramedullary nail bends, absorbing much of the energy of the trauma. Also more energy is required to cause a refracture and bending of an intramedullary nail in completely healed fractures than in incompletely healed [5]. 

The first step in approaching fractures recurrences is the well-planned attempt to remove the deformed intramedullary nail. Removing a bent intramedullary nail is very difficult and requires strategy and inspiration. Few methods have been described in literature such as (a) in situ straightening via external force on the femur [4], (b) the sectioning of the nail and removal of each piece separately [2], and (c) sectioning the nail to half its diameter and then breaking it [3].

Reviewing the available literature reveals that each case of distorted intramedullary nail removal was approached uniquely [1–5]. Technical support played a vital role in the method used to removal the nail with the least amount of damage to bone and soft tissue. 

In our case and after much deliberation, it became evident that technical support was unable to give viable solutions to our problem. Sterilized diamond-edged blades for the sectioning of the intramedullary nail were unavailable. After much thought it was decided to apply a different strategy to the removal of the intramedullary nail. 

## 5. Conclusion

Refracture of the femur shaft with an in situ intramedullary nail following a high-velocity trauma is rare. Nail removal is difficult if the intramedullary nail is bent or distorted. There is a high risk of infection and delayed union or nonunion of the fracture due to multiple damage of the soft tissue envelope and disruption of blood supply. However, dealing with such cases is a great challenge for the orthopaedic surgeon due to the fact that every such incident requires a unique therapeutic and surgical approach.
